# Wnt4 coordinates directional cell migration and extension of the Müllerian duct essential for ontogenesis of the female reproductive tract

**DOI:** 10.1093/hmg/ddv621

**Published:** 2015-12-31

**Authors:** Renata Prunskaite-Hyyryläinen, Ilya Skovorodkin, Qi Xu, Ilkka Miinalainen, Jingdong Shan, Seppo J. Vainio

**Affiliations:** 1 Oulu Centre for Cell-Matrix Research, Faculty of Biochemistry and Molecular Medicine, Biocenter Oulu, Laboratory of Developmental Biology, InfoTech Oulu, University of Oulu, PO Box 5000, FIN-90014 Oulu, Finland and; 2 Biocenter Oulu, University of Oulu, 90220 Oulu, Finland

## Abstract

The Müllerian duct (MD) is the anlage of the oviduct, uterus and upper part of the vagina, the main parts of the female reproductive tract. Several wingless-type mouse mammary tumor virus (MMTV) integration site family member (Wnt) genes, including *Wnt4*, *Wnt5a* and *Wnt7a*, are involved in the development of MD and its derivatives, with *Wnt4* particularly critical, since the MD fails to develop in its absence. We use, here, *Wnt4^EGFPCre^*-based fate mapping to demonstrate that the MD tip cells and the subsequent MD cells are derived from Wnt4+ lineage cells. Moreover, *Wnt4* is required for the initiation of MD-forming cell migration. Application of anti-Wnt4 function-blocking antibodies after the initiation of MD elongation indicated that Wnt4 is necessary for the elongation as well, and consistent with this, cell culture wound-healing assays with NIH3T3 cells overexpressing *Wnt4* promoted cell migration by comparison with controls. In contrast to the *Wnt4* null embryos, some *Wnt4^monomeric cherry/monomeric cherry^* (*Wnt4^mCh/mCh^*) hypomorphic mice survived to adulthood and formed MD in ∼45% of cases. Nevertheless, the MD of the *Wnt4^mCh/mCh^* females had altered cell polarization and basement membrane deposition relative to the controls. Examination of the reproductive tract of the *Wnt4^mCh/mCh^* females indicated a poorly coiled oviduct, absence of the endometrial glands and an undifferentiated myometrium, and these mice were prone to develop a hydro-uterus. In conclusion, the results suggest that the *Wnt4* gene encodes signals that are important for various aspects of female reproductive tract development.

## Introduction

The mammalian sex ducts are formed from the female Müllerian duct (MD) and the male Wolffian duct (WD) during embryogenesis. The paired MD, also called the paramesonephros, represents the primordium of the oviduct, the uterus and the upper part of the vagina, and is an integral part of the embryonic urogenital system. The MD initially forms in both the male and the female, but degenerates later in the male under the influence of the anti-Müllerian hormone (1,2).

The MD was described more than 200 years ago, but there are still lively discussions going on even today about its origin. It is now well-established that the MD cells do not originate from the WD cell population (2–4), even though the WD does provide important mechanical guidance cues and secretes certain signals that promote MD development (5). It is known that the coelomic epithelium (CoE) contributes to MD development, but it is unclear (in Amniota) whether it is invagination or a local thickening of CoE that establishes the primordium of the MD (3,4). What controls and guides the posterior elongation of the MD is also an open question.

Gene targeting experiments have indicated that several factors such as *Gata3*, paired-box gene 2 (*Pax2*), paired-box gene 8 (*Pax8*), LIM homeobox protein 1 (*Lhx1*)*,* empty spiracles homeobox 2 (*Emx2*), homeobox A13 (*Hoxa13*) and wingless-type mouse mammary tumor virus (MMTV) integration site family member 4 (*Wnt4)*, *Wnt7a, Wnt9b* regulate MD development (5–14). Of these, the *Wnt4* gene encodes one of the key signals, since the MD fails to develop in its absence and only the extreme anterior *Wnt7a*-positive primordium is formed (13). Otherwise, the role of the *Wnt4* gene during MD development remains unclear.

The MD-derived oviduct, uterus and upper part of the vagina reach sexual maturity around puberty. The mature uterus is composed of the endometrial stromal cells and the myometrium, which has inner and outer layers. During postnatal development the endometrial glands, which provide nutrients, growth factors and cytokines to prepare the uterus for possible pregnancy are derived from the luminal epithelium. Failure of these sequential steps is connected with infertility. Mutations in the human *WNT4* gene are associated with Mayer–Rokitansky–Kuster–Hauser–Biason–Lauber (MRKHBL) and female SEx Reversal and dysgenesis of Kidneys, Adrenals, and Lungs (SERKAL) syndromes, which involve severe defects in the female reproductive tract but the underlying molecular mechanisms that distinguish between a normal and a pathological uterus are still for the most part poorly understood (15–20).

We have been able to show by means of time-lapse organ culture that the *Wnt4*+ progenitor cells contribute to the MD primordium and represent an important cell population that is required for MD assembly. Our findings also demonstrate that *Wnt4* is needed not only for initiation of MD-forming cell migration and tip cell differentiation, but also for MD elongation. They suggest that the MD initiates its growth from the coelomic epithelial cells that invade the space beneath it and establish a funnel-shaped MD progenitor cellular unit. Moreover, using a novel hypomorphic *Wnt4 monomeric cherry* (*Wnt4mCh*) mouse model we have shown that *Wnt4* is needed for cell polarization and proper basement membrane (BM) deposition in the developing MD and that it is also required later in uterine ontogenesis for endometrial gland and myometrium organization. Thus, the hypomorphic *Wnt4mCh* mice serve as a useful model for studying the mechanisms lying behind hyperplastic MD malformations and agenesis. The results suggest that *Wnt4* may be involved in the development of endometrial disease and female infertility, thereby extending the role of this female sex determinant as a signal for the ontogenesis of the female reproductive tract.

## Results

### Initiation of MD growth requires *Wnt4* signalling

The MD fails to form in *Wnt4* knock-out embryos and only the anterior MD precursor cells differentiate, as depicted by the *Wnt7a* MD marker in *in situ* hybridization (Supplementary Material, Fig. S1, compare B with A, arrow) (13). To understand better how *Wnt4* coordinates MD development, we examined the location of lineages derived from cells that have expressed *Wnt4* by making the use of *Wnt4^EGFPCre^* mice crossed with floxed *RosaR26R LacZ* (*R26R LacZ*) so that the *LacZ* gene was flanked by *LoxP* sites and became activated by the *Cre* reaction.

Although no *LacZ* staining was seen in the controls at any of the stages analysed (Supplementary Material, Fig. S1C, E, G and I), the *Wnt4^EGFPCre^; R26R^LacZ^* embryos had positive cells scattered within the urogenital ridge at E12.5-E15.5 (Supplementary Material, Fig. S1D, F, H and J). These were more numerous in the anterior side of the urogenital ridge at E12.5 (Supplementary Material, Fig. S1D, arrow), whereas later, at E14.5, they tended to accumulate in the posterior portion (Supplementary Material, Fig. S1H, arrow).

Such observations led us to hypothesize that *Wnt4* may be a factor that controls MD development by promoting cell migration. To study this, we performed lineage tracing of the cells expressing *Wnt4* by time-lapse imaging of *ex vivo* urogenital ridge explant cultures from *Wnt4^EGFPCre^*; *RosaR26R YFP (R26R YFP)* embryos (Supplementary Material, Movies S1 and S2).

The movies revealed *YFP* expression in the region of the mesonephric tubules of the urogenital ridge, but not in the WD (Supplementary Material, Fig. S2A–F and Movie S1). Moreover, the *YFP*+ cells started to migrate from the anterior region towards the posterior region laterally with respect to the WD and parallel to it (arrows in Supplementary Material, Fig. S2A–F and Movie S1). Urogenital ridges of *Wnt4^EGFPCre^* embryos served as control material (Supplementary Material, Fig. S2G–L and Movie S2). This migration of the *YFP*+ cells correlated with the formation of the MD and occurred in both male and female embryonic explants.

The *YFP*+ cells migrated along the WD and CoE and appeared to shape the MD in the urogenital ridges (Supplementary Material, Movies S1). They then continued their migration along the resulting MD primordium even after it had reached the urogenital sinus (Supplementary Material, Movies S1). We conclude that cell lineages defined by *Wnt4* expression participate in growth of the MD.

To characterize the spatial and temporal organization of the MD with respect to the WD and the CoE, we next performed anti-Pax2 immunostaining of the urogenital ridges at the age of 23–26 tail somites. The staining depicted both MD and WD structures (Supplementary Material, Fig. S2M–O). Further optical projection tomography (OPT) then provided 3D evidence of the funnel-shaped appearance of the anterior part of the MD and its close proximity to the WD at E12.5 (Supplementary Material, Fig. S2P, arrows).

### The MD tip cells and some coelomic epithelial cells are derived from cells expressing Wnt4

For a better insight into the dynamics of how *Wnt4*+ lineage cells participate in MD generation, we set out to obtain confocal time-lapse images of the *Wnt4^EGFPCre^; mT/mG*-positive embryonic urogenital ridge explants. In the *mT/mG* reporter, a membrane-targeted tomato (*mT*, red) is normally expressed in all the urogenital ridge cells. Once recombined with the *Wnt4^EGFPCre^* the cells start to express cell membrane-targeted green fluorescence protein (*mG*) and are considered to be the *Wnt4*+ lineage cells.

**Figure 1.**
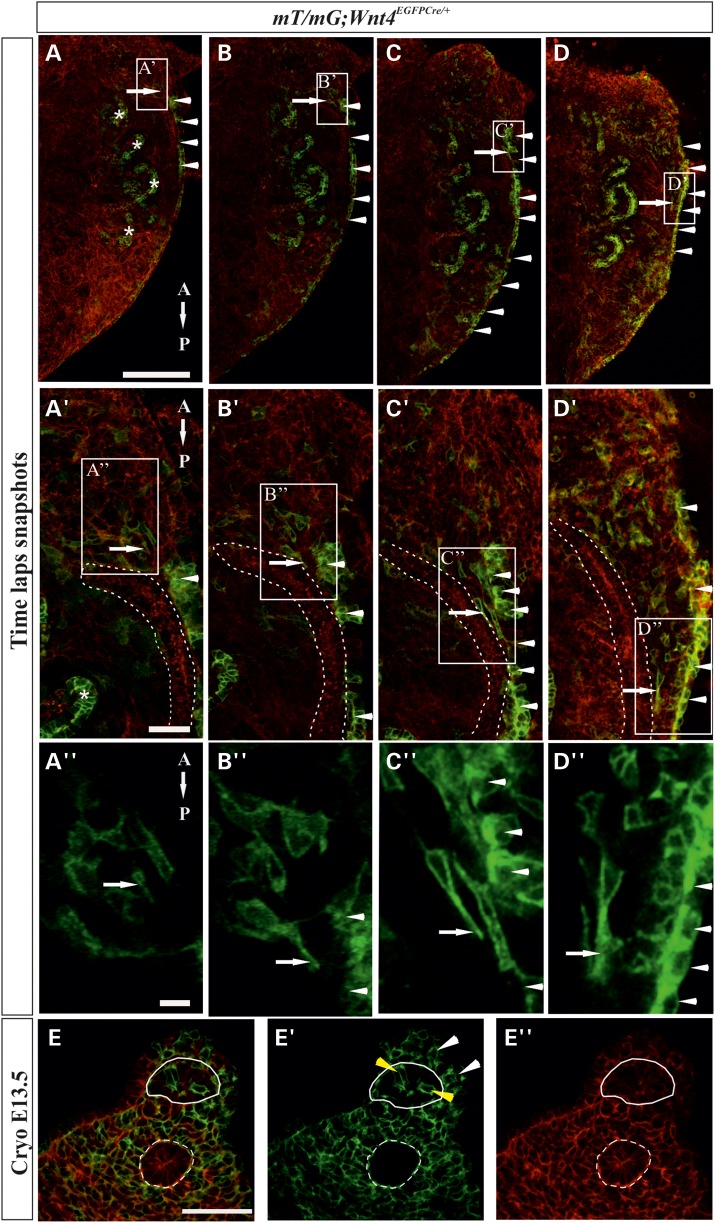
The MD is constructed of cells of *Wnt4^EGFPCre^* lineage via cell migration, as depicted in time-lapse imaging. (**A**–**D**″) Selected snapshots from the time-lapse movie presented in Supplementary Material, Movie S3 depict an organ culture of a *Wnt4^EGFPCre^*; *mT/mG* embryonic urogenital ridge. (A) The MD primordium forms in the anterior part of the urogenital ridge in close proximity to the WD. Note the GFP+ *Wnt4* lineage cells (A′ and A″ arrows). The CoE serves as a landmark for the migrating MD-forming cells (A′–D″, arrowhead). (B–B″) Micrographs depict a cell located in the MD primordium that has generated extensions (arrow) between the WD and the CoE (arrowhead). (C–C″) After differentiation of the tip cells (arrow) the MD-forming cells start to migrate in a posterior direction. (D–D″) The cellular processes protrude though the extracellular matrix between the WD and the CoE, which is positive for GFP+ (arrowheads). (**E**–E**′″**) Cryo-sections of the genital ridges of *Wnt4^EGFPCre^*; *mT/mG* embryos. (E) Merged images of the GFP+ (green) and mTomato (red) fluorescent cells marking *Wnt4* lineage. (E′) The GFP+ cells of *Wnt4+* lineage are seen (in green). The yellow arrowheads point to the *Wnt4+* lineage cells contributing to MD assembly. Note that, there are also *GFP−* cells among the MD cells. Cells of *Wnt4+* lineage are also present in the CoE, while the WD is negative for GFP. (E′″) Hoechst staining depicts the nuclei of the cells. The dotted line underlines the WD and the solid line the MD. Scale bars (A–D) 250 µm, (A′–D′) 50 µm, (A″–D″) 10 µm and (E–E′″) 50 µm.

The time-lapse imaging revealed a specific group of GFP+ cells that formed the MD primordium (Fig. 1A–A″, arrows and Supplementary Material, Movie S3). It is significant that GFP was also expressed in the CoE (Fig. 1A′–D′, arrowheads) and the mesonephric tubules (Fig. 1A′–D″star), but not in the WD (Fig. 1A′–D″ and E, dashed line).

We observed both GFP+ and GFP− cells that moved beneath the CoE and on the anterior side of it, and laterally with respect to the WD. Based on these data we define this heterogenous cell population as representing the MD node in the urogenital ridge (Supplementary Material, Movie S3). After several hours of culture, the GFP-positive cells derived from the MD node were seen to develop cytoplasmic extensions (Fig. 1A″–D″, arrows) and to protrude between the WD (Fig. 1A″–D″, dashed line) and the CoE (Fig. 1A″–D″, arrowheads). The migratory stream of GFP+ and GFP− cells behind the leading GFP+ cells also took part in shaping the MD primordium (Fig. 1A′–D′ and Supplementary Material, Movie S3). The CoE cells turned the *GFP* expression on via *Wnt4^EGFPCre^* in correlation with the process of MD elongation in developing urogenital ridge (Fig. 1A–D and Supplementary Material, Movie S3).

After the MD primordium had extended and reached the developing urogenital sinus, other cells continued to migrate towards the urogenital sinus via the maturing MD (Supplementary Material, Movie S3). Interestingly, some cells forming the MD were capable of changing their direction of migration and reverted from posterior movement to migrate anteriorly, against the dominant stream (Supplementary Material, Movie S3).

To depict the localization of the GFP+ cells during MD development, we made cryostat cross-sections of the *Wnt4^EGFPCre^; mT/mG* (Fig. 1E–E″′) and control (Supplementary Material, Fig. S2Q–Q′″) urogenital ridges at E13.5. The CoE and mesenchyme surrounding the developing MD and WD contained GFP+ cells (Fig. 1E′ white arrows), and some cells in the epithelial MD were GFP+ (Fig. 1E′, yellow arrow heads), whereas no *GFP* expression was noted in the WD (Fig. 1E–E″).

The presence of Wnt4+ lineage cells in the CoE alongside the elongating MD primordium suggested that the coelomic epithelium might play a role in MD growth. We made a mechanical incision in the CoE and studied the outcome by time-lapse imaging. Analysis of the movies showed that growth of the MD terminated at the level of the mechanical incision in the CoE and that the tip cells also made several attempts to migrate beyond the incision site, but failed (Supplementary Material, Movie S4 and Fig. S2R).

In order to reliably assign the failure in cell migration to the CoE, we analysed the integrity of the WD via confocal sections visible in the individual frames of the time-lapse movies. The results revealed that the mechanical insult was present only in the CoE and suggested an important role for the CoE in MD development. The study also indicated that the *Wnt4* signal is critical for MD tip cell specification and for the initiation of migration of the MD-forming cells.

### The *Wnt4* signal is important for elongation of the MD

The data raised the question of whether *Wnt4* would have a role in the subsequent growth of the MD. To study this possibility, we used anti-Wnt4 antibodies to inhibit Wnt4 activity in urogenital ridge explants from E11.5 embryos in an organ culture. The control specimens were treated with goat anti-human IgG. After 48 h of culture the explants were fixed, stained with *Wnt7a* RNA probe and analysed.

**Figure 2.**
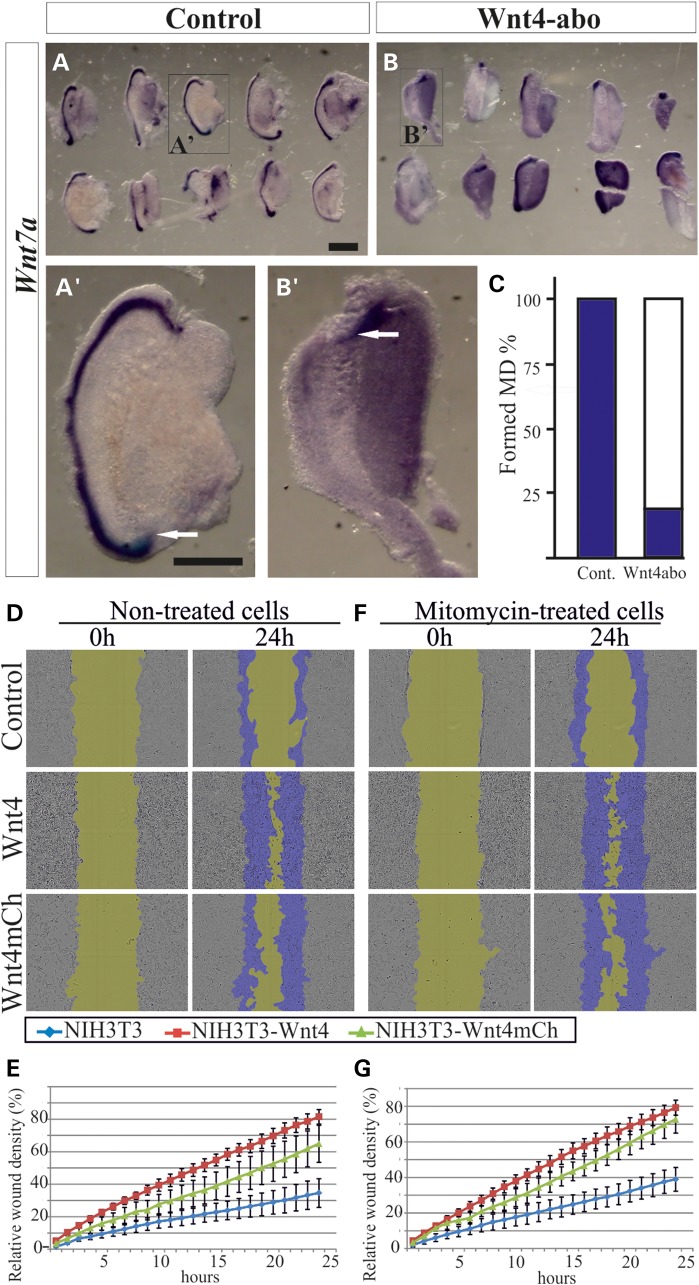
The *Wnt4* function is required for the extension of the MD and scratch-wound recovery *in vitro*. Urogenital ridges were dissected at E11.5 and grown for 48 h in the presence of goat IgG immunoglobulins as controls (**A**). The MD was detected with the *Wnt7a* marker (A, and enlarged in **A**′, arrow). The MD failed to develop in ∼79% of the cases upon supplementation with anti-Wnt4 antibody (**B**, and enlarged in **B**′, arrow) (**C**). (**D**) Micrographs from the *in vitro* wounding assay with control NIH3T3, NIH3T3*Wnt4+* and NIH3T3*Wnt4^mCh^*^/*mCh+*^ cells reveal a positive influence of *Wnt4* on wound closing, as quantified in (**E**). (**F**) Treatment of the cells with mitomycin did not alter the behaviour of these cells significantly, as quantified in (**G**). Scale bars (A, B and A′–B″) 500 µm.

In the presence of the control IgGs the MD grew and reached the posterior end of the WD in all the samples studied (24/24) (Fig. 2A, enlarged in A′), whereas MD growth was inhibited in 79% (19/24) of cases in the presence of the anti-Wnt4 antibody (Fig. 2B, enlarged in B′), but the MD did develop in the remaining 21% (5/24) (Fig. 2C). We conclude that *Wnt4* signalling is required not only for initiation of the cell migration that leads to MD formation, but also for MD extension.

### *Wnt4* promotes cell migration *in vitro*, as judged by cell scratch-wound assay

Since the time-lapse studies of the *Wnt4^EGFPCre^*+ cells in the urogenital ridge explants suggested a role for *Wnt4* in the control of cell migration, we analysed this further by means of *in vitro* scratch-wound assays of NIH3T3 cells that expressed *Wnt4* and *Wnt4mCh*, the latter being a fusion between *Wnt4* and the *mCherry* protein, representing a hypomorphic allele (21). The parental NIH3T3 cells, which did not express Wnt4 protein (22), were used as a control.

In order to determine whether scratch-wound closure was driven by cell proliferation or cell migration properties, we treated one set of cells with mitomycin, an inhibitor of cell proliferation. After wounding, the cells were cultured and imaged once per hour for 24 h. We then analysed the resulting time-lapse images and calculated the relative scratch-wound density after 24 h of culture (Fig. 2D).

The relative wound density was 42% in the control cells, 74% in the NIH3T3*Wnt4* cells and 67% in the NIH3T3*Wnt4mCh* cells (Fig. 2E). Similar results were obtained in the presence of mitomycin (Fig. 2F). The relative scratch-wound densities were close to those for untreated cells after 24 h of culture, which were 54% in the control cells, 72% in the NIH3T3*Wnt4* cells and 59% in the NIH3T3*Wnt4mCh* cells (Fig. 2G). Taken together, the data suggest that *Wnt4* may control MD development by inducing cell migration.

### The canonical Wnt signalling pathway inhibiting small molecule XAV939 influences MD formation

As *Wnt4* may signal via both the canonical/β-catenin pathway and non-canonical pathways (23–25), we used the tankyrase inhibitor XAV939 to ascertain whether β-catenin-mediated signalling is involved in MD development (26). The XAV939 was diluted in dimethyl sulphoxide (DMSO) and control specimens were supplemented with media containing a corresponding amount of DMSO alone. After 48 h of culture, the degree of MD development was evaluated by *in situ* hybridization with the *Wnt7a* probe.

**Figure 3.**
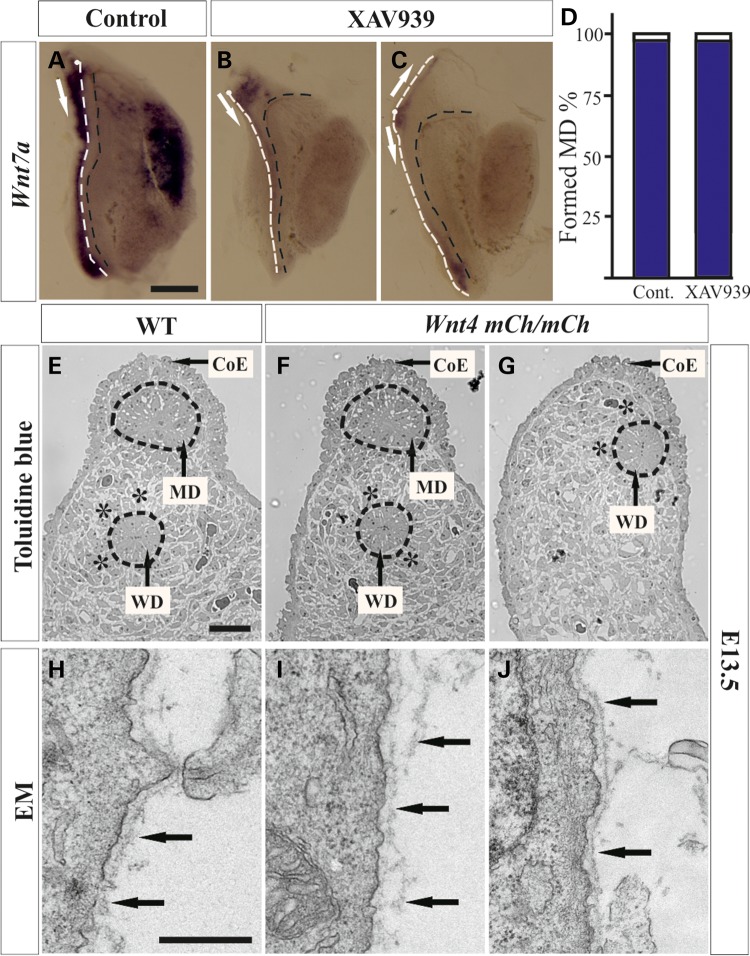
Changes in MD growth caused by the tankyrase inhibitor XAV939 and the hypomorphic *Wnt4^mCh^*^/^*^mCh^* allele. The MD develops normally in the presence of DMSO, serving as a control (**A**, white line, arrow). The *Wnt7a* MD marker is weak in the presence of XAV939, so that ∼41% of the MDs have branched on the apical side (**B**, arrows) and some have extended not only posteriorly, but also anteriorly (**C**, arrows). XAV939 does not block MD growth, but it severely affects the properties of the resulting MD (**D**). (**E**) Toluidine blue staining detects the wild-type urogenital ridge at E13.5. The MD was formed in 45% of the *Wnt4^mCh^*^/*mCh*^ mice (**F**) and failed to form in 55%. The mesenchymal cells were not organized in a concentric manner around the WD at E13.5 in the *Wnt4^mCh^*^/*mCh*^ mice, in contrast to the controls (stars in E and G). The failure of MD formation influences the localization of the WD and CoE (arrows in E and G). The transmission electron micrographs depict a well-condensed BM around the control MD (**H**, arrows) but a loose, malformed one around its *Wnt4^mCh^*^/*mCh*^ counterpart (**I**, arrows). The BM surrounding the WD remains intact (**J**, arrows). MD, white dashed line, and WD, black dashed line in (A–C). CoE, coelomic epithelium. Scale bars (A–G) 500 µm and (H–J) 250 nm.

MD growth was initiated and the duct had extended posteriorly in both the control urogenital ridge specimens (96%) and those supplemented with XAV939 (95.8%) although the latter had an ambiguous MD and only weak *Wnt7a* RNA probe staining (Fig. 3A and B). In some cases, the MD extended not only posteriorly, as in the non-treated specimens, but also more apically (Fig. 3C). We conclude that the β-catenin signalling pathway inhibitor XAV939 does not perturb MD growth, but it does influence the overall MD morphology *in vitro*.

### The hypomorphic allele depicts roles for *Wnt4* in reproductive tract development during embryogenesis

To address the putative later functions of *Wnt4* in the development of the urogenital ridge, we took advantage of the recently developed novel *Wnt4^mCh/mCh^* mouse model, which represents a fusion protein and provides a hypomorphic *Wnt4* allele (21).

Interestingly, the MD was found to be formed in ∼45% of the *Wnt4^mCh/mCh^* embryos, but in the remaining 55% it had failed to develop (Fig. 3E–G). This made it possible to study the role of *Wnt4* later on in sex-duct development in more detail. We analysed the degree of MD development in the urogenital ridge at three time points: E13.5, when the MD has just formed, E14.5 when it is still undifferentiated, and before birth, at E18.5, when it has become differentiated into the oviduct, uterus and upper part of the vagina.

The *Wnt4^mCh/mCh^* MD phenotype varied from being close to the wild-type to complete ductal agenesis, as depicted at E13.5 (Fig. 3E–G). The lack of any *Wnt4* signal also affected the CoE (Fig. 3E, arrows). In the cases where the duct did not form, the CoE cells became cuboidal and were displaced together with the WD (Fig. 3G, compare with E). The mesenchymal cells normally establish a concentric organization around the WD in wild-type urogenital ridges, whereas the *Wnt4^mCh/mCh^* mesenchymal cells failed to do so. The defect was more prominent in those urogenital ridges where the MD did not form at all (Fig. 3G, compare with E and F stars).

**Figure 4.**
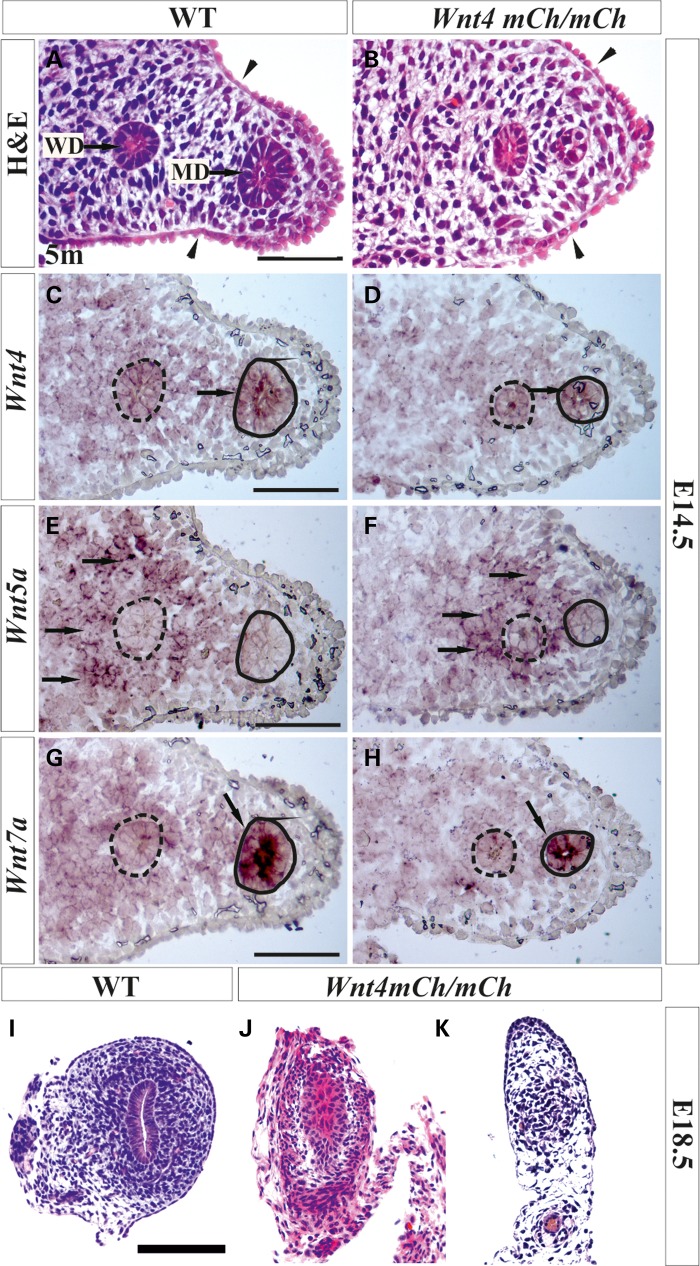
The *Wnt4^mCh^*^/*mCh*^ hypomorph influences prenatal development of the MD and uterus. (**A**) The wild-type mice have a well-developed MD at E14.5, whereas MD formation is impaired in the *Wnt4^mCh^*^/*mCh*^ mice (**B**). The arrowheads in (A and B) depict the urogenital ridge groove that typically fails to form in the *Wnt4^mCh^*^/*mCh*^ embryos (compare B with A). The *Wnt4* gene is expressed in the MD (**C** and **D**, arrow) and in the mesenchyme around the MD and the WD in both the control (C) and *Wnt4^mCh^*^/*mCh*^ embryos (D). *Wnt5a* is expressed in the mesenchyme (**E** and **F**, arrows), while *Wnt7a* mRNA is located in the epithelial cells of the MD (E and **G**, respectively, arrows) in the wild-type and *Wnt4^mCh^*^/*mCh*^ embryos (F and **H**, arrows). (**I**) The wild-type uterus has an open lumen at E18.5, whereas the *Wnt4^mCh^*^/*mCh*^ embryos have a rudimentary uterus (**J**) or none at all (**K**). The continuous line depicts the MD and the dashed line the WD (C–H). Scale bars (A–H) 50 µm, (E–G) 100 µm.

Examination of the ultrastructure of the *Wnt4^mCh/mCh^* MD cells revealed that the BM was slack in its deposition at E13.5 and formed loops, whereas the wild-type BM was more solid (Fig. 3H compare with I, arrows). In contrast, the WD had a properly developed BM in both the wild-type (data not shown) and the *Wnt4^mCh/mCh^* specimens (Fig. 3J, arrows). The grooves of the MD ridge, as seen in the wild-type, were often missing in the *Wnt4^mCh/mCh^* embryos, as shown at the age of E14.5 (Fig. 4A and B, arrowheads). The MD differed notably in diameter between the *Wnt4^mCh/mCh^* and control specimens (Fig. 4A and B).

*In situ* hybridization analysis of *Wnt4*, *Wnt5a* and *Wnt7a*, the Wnt genes involved in MD development, indicated that the MD and its surrounding stromal cells in both the wild-type and the *Wnt4^mCh/mCh^* embryos were positive for the *Wnt4* RNA probe at E14.5 (Fig. 4C and D). There was extensive *Wnt5a* expression in the stroma of the urogenital ridge around the WD in the controls, whereas the expression was more compact in the *Wnt4^mCh/mCh^* mice (Fig. 4E and F). *Wnt7a* was expressed in both the control and *Wnt4^mCh/mCh^* epithelial cells, (Fig. 4G and H). We conclude that the reduction in *Wnt4* signalling does not alter the expression of the *Wnt5a* or *Wnt7* genes in the urogenital ridge to any great extent.

The wild-type uterus was robust and oviduct had formed folds at E18.5 (Supplementary Material, Fig. S3A and B), whereas the uterus was ambiguous and the oviducts lacked folds in the *Wnt4^mCh/mCh^* embryos (Supplementary Material, Fig. S3C and D). Histological sections of the wild-type embryo uterus exhibited a well-defined, open uterine lumen (Fig. 4I), while the uterus in the *Wnt4^mCh/mCh^* embryos was either rudimentary (Fig. 4J) or had completely failed to form (Fig. 4K).

### *Wnt4* signalling is critical for formation of the endometrial glands of the uterus

**Figure 5.**
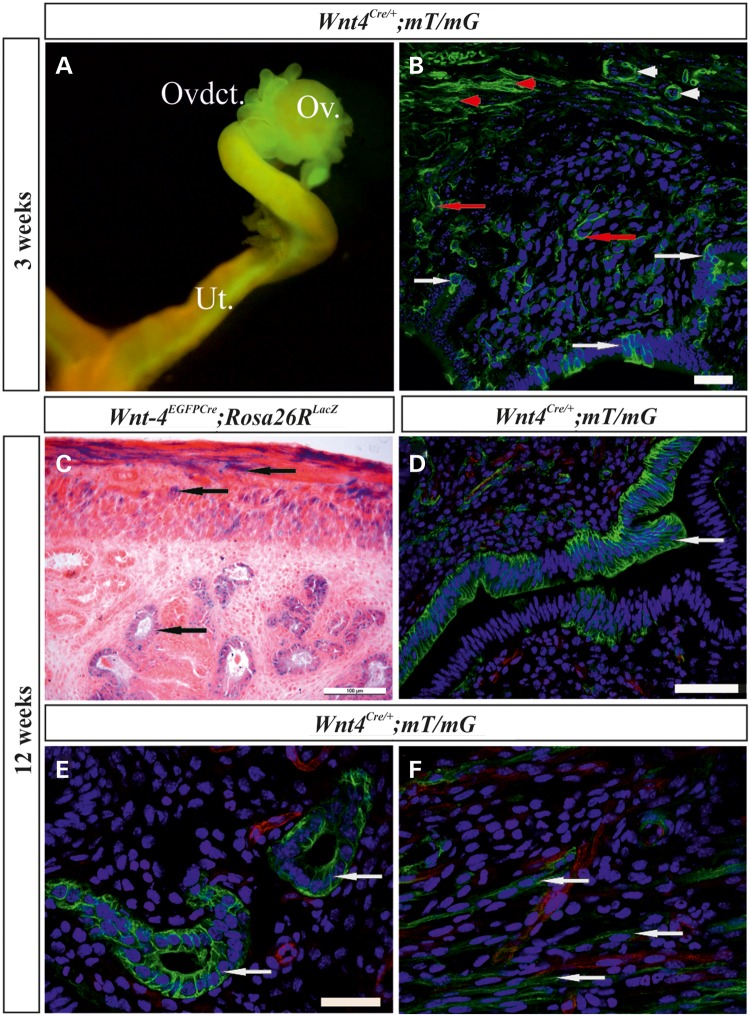
Fate mapping of the established *Wnt4^EGFPCre^* progenitor cell lineage reveals daughter cells in the female reproductive tract. (**A**) Marked GFP+ cells are detected in the ovary (Ov), oviduct (Ovdct) and uterus (Ut) of the *Wnt4^EGFPCre^*;*mT/mG* mice at 3 weeks of age (P21). (**B**) GFP+ cells are situated in the uterine luminal epithelium, the luminal glands (white arrows) and the interstitial (red arrows), myometrium (red arrowheads) and endothelial cells around the blood vessels (white arrowheads). (**C**) β-galactosidase staining of the uterus of the *Wnt4^EGFPCre^; RosaR26RLacZ* females reveals positive cells in the myometrium and endometrial glands (arrows). (**D**) Marked *GFP*+ cells are present in the epithelium of the uterus (arrow), the endometrial glands (**E**, arrows) and the myometrium (**F**, arrows) of the *Wnt4^EGFPCre^*;*mT/mG mice* at 12 weeks of age (C–F). Scale bar (B–F) 100 µm.

Analysis of the distribution of *Wnt4*+ lineage cells in the uterus of postnatal *Wnt4^EGFPCre^; mT/mG* mice at 3 weeks of age (postnatal day P21, Fig. 5A) showed these to be present in the uterine epithelium (Fig. 5B, white arrows), luminal glands (Fig. 5B, red arrows), myometrium (Fig. 5B, red arrowheads) and endothelial cells surrounding the blood vessels (Fig. 5B, white arrowheads). A corresponding analysis performed on *Wnt4^EGFPCre^; RosaR26R^LacZ^* mice showed that cells of *Wnt4+* lineage were located among the myometrium cells of the uterus (Fig. 5C, black arrows) and in the endometrial gland epithelium (Fig. 5C, white arrows).

The 12-week-old *Wnt4^EGFPCre^*;*mT*/*mG* mice showed *GFP*-positive cells in the luminal epithelium (Fig. 5D, arrow), endometrial glands (Fig. 5E, arrow) and myometrium of the uterus (Fig. 5F, arrows). In conclusion, cells of *Wnt4+* lineage participate in MD growth and contribute later to the formation of the uterine myometrium and endometrium and to its vasculature development.

**Figure 6.**
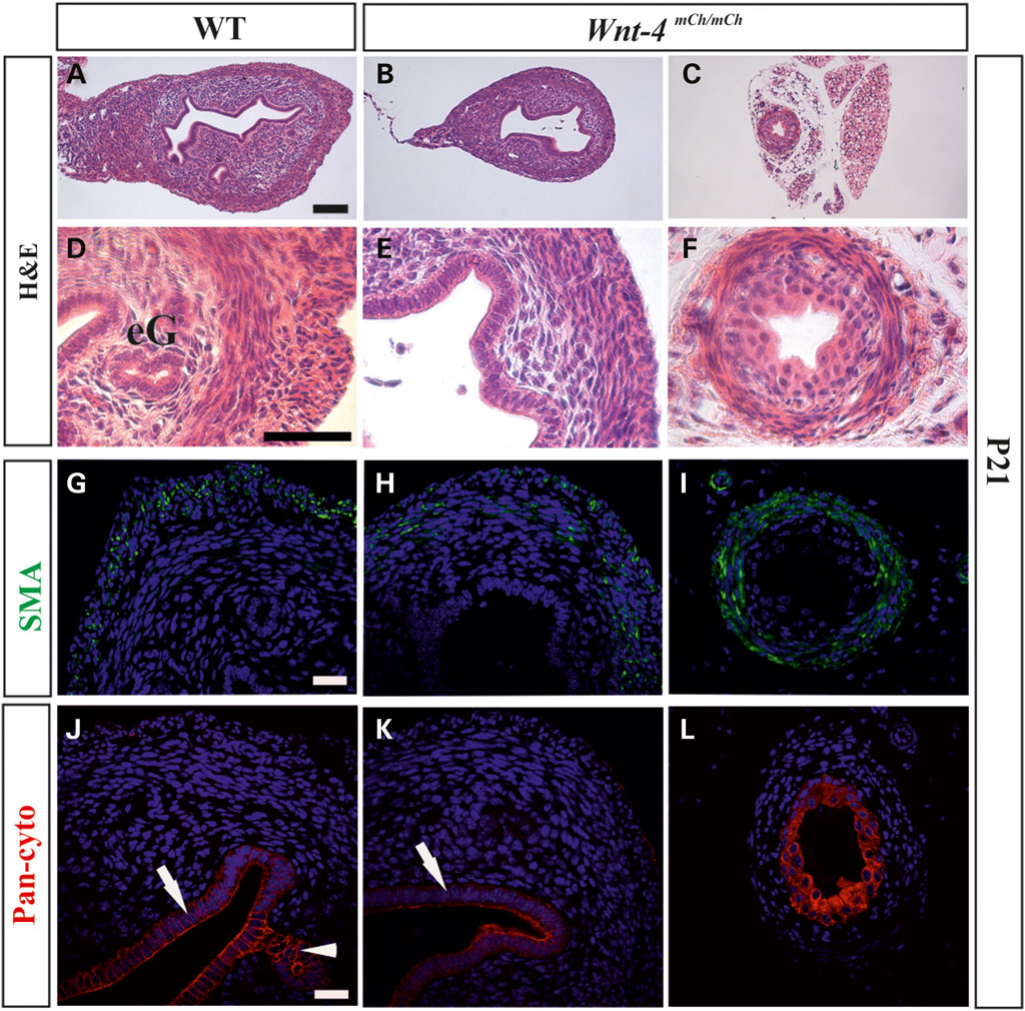
The uterus is severely compromised in adult hypomorphic *Wnt4^mCh^*^/*mCh*^ females. The uterus is reduced in size in *Wnt4^mCh^*^/*mCh*^ adult mice (P21) and no luminal or endometrial glands are formed, whereas these develop in the wild-type mice (compare **B** and **E** with **A** and **D**). (**C** and **F**) In some cases the WD derivatives remained in the *Wnt4^mCh^*^/*mCh*^ females and formed a vas deferens-like structure (C and F). SMA (green) staining depicts two distinct muscle layers in the wild-type uterus at P21 (**G**), whereas the *Wnt4^mCh^*^/*mCh*^ uterus has a thinner myometrium layer (**H**). The vas deferens-like structure in the *Wnt4^mCh^*^/*mCh*^ females has a smooth muscle distribution typical of a wild-type male (**I**). (**J**) Pan-cytokeratin staining highlights the luminal epithelium and forming luminal glands (arrow) in the wild-type, whereas no luminal glands have formed in the *Wnt4^mCh^*^/*mCh*^ mice at 5 months of age (**K**, arrow). (**L**) The epithelium of the vas deferens-like structure in the *Wnt4^mCh^*^/*mCh*^ mice had a distorted cell distribution, as depicted by pan-cytokeratin staining (P21). Scale bars (A–C) 100 µm, (E and F) 50 µm and (G–L) 100 µm.

Further analysis of the uterus before sexual maturity, on Day P21, demonstrated that the *Wnt4^mCh/mCh^* mice had a thinner uterus than the wild-type mice (Fig. 6B and E compare with A and D). Notably, in line with *Wnt4* knock-out females that become masculinized (13), the *Wnt4^mCh/mCh^* female mice had WD derivatives, an epididymis (21) and a vas deferens type of structure embedded in fat-like tissue (Fig 6C and F). Staining with alpha-smooth muscle actin (α-SMA) antibody showed smooth muscle cell differentiation in both the wild-type and the *Wnt4^mCh/mCh^* uterus (Fig. 6G and H), and a smooth muscle layer had also formed in the vas deferens-like tube (Fig. 6I).

The pan-cytokeratin antibody had stained the luminal epithelium and the emerging endometrial glands in the wild-type, but importantly, no endometrial gland formation was detected in the *Wnt4^mCh/mCh^* uterus (Fig. 6J and K). The vas deferens-type structure had epithelial cells that were positive for pan-cytokeratin staining, but they were cuboidal in shape rather than pseudo-stratified and columnar as is typical of the male vas deferens (Fig. 6L).

Some of the *Wnt4^mCh/mCh^* mice reached sexual maturity and survived to 5 months of age, but most of them had unilateral or bilateral hydro-uterus, probably caused by the congenital vaginal abnormalities (Supplementary Material, Fig. S3E compare with F and G). In the cases where the uterus had failed to form, only a primitive mesometrium was present (Supplementary Material, Fig. S3H, arrow heads).

**Figure 7.**
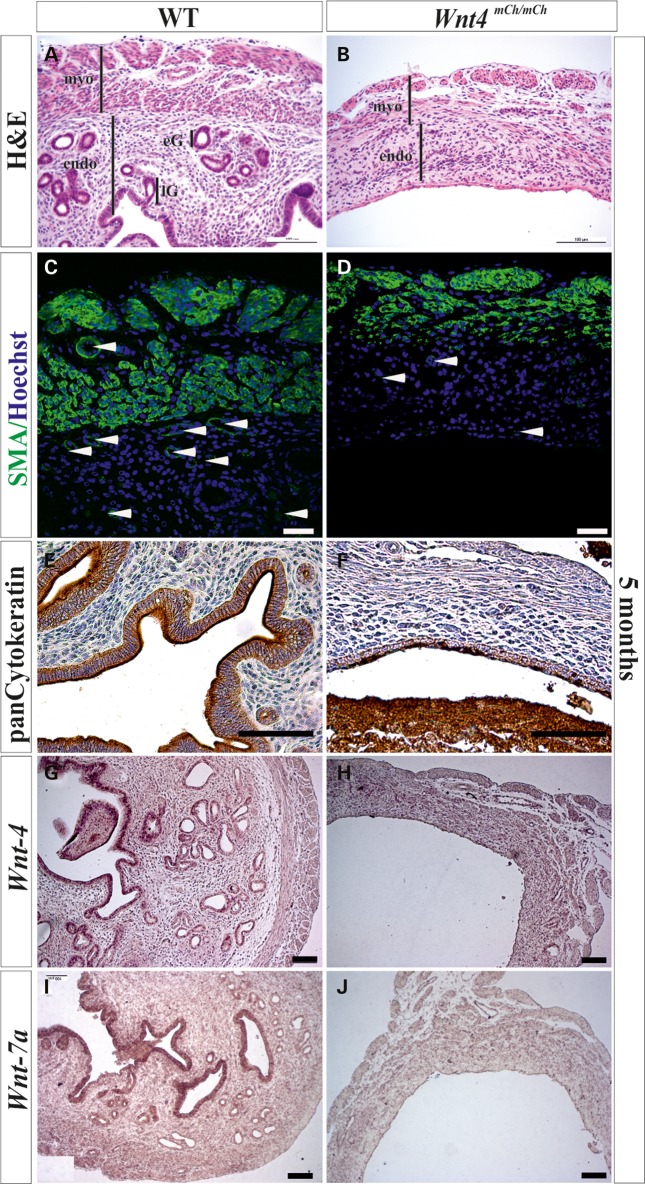
The myometrium and endometrium of the uterus are compromised in *Wnt4^mCh^*^/*mCh*^ females. (**A**) The wild-type uterus has a well-differentiated myometrium and endometrium with endometrial glands. (**B**) The *Wnt4^mCh^*^/*mCh*^ uterus has a thin layer of myometrium and endometrium without any luminal epithelium or endometrial glands. (**D**)The uterus normally has two layers of smooth muscle cells and a well-vascularized endometrium (**C**, arrowheads), whereas the *Wnt4^mCh^*^/*mCh*^ uterus fails to form two layers within the myometrium and the number of blood vessels is reduced (compare D with C, arrowheads). Pan-cytokeratin antibody staining shows the formation of endometrial glands in the wild-type mice (**E**) and failure of endometrial gland formation in the rudimentary epithelium of the *Wnt4^mCh^*^/*mCh*^ mice (**F**). (**G**) The *Wnt4* gene is expressed normally in the luminal epithelium and endometrial glands of the wild-type uterus, but is lost in the case of *Wnt4^mCh^*^/*mCh*^ hypomorphism (compare **H** with **G**). (**I**) *Wnt7a* is expressed in the luminal epithelium and luminal glands of the wild-type uterus, but no mRNA is detected in the *Wnt4^mCh^*^/*mCh*^ uterus (**J**) at 5 months of age. Myo, myometrium; endo, endometrium; eG, endometrial gland; lG, luminal glands. Scale bar (A–J) 100 µm.

Histological examination revealed that while the wild-type uterus had a clearly distinguishable myometrium, endometrium and lumen (Fig. 7A), the uterus in the severe cases among the *Wnt4^mCh^*^/*mCh*^ mice was dilated and largely lacking in uterine components (Fig. 7B). The smooth muscle layer of the uterine myometrium is normally composed of two parts, an inner circular one and an outer longitudinal one (Fig. 7C), but in the *Wnt4^mCh^*^/*mCh*^ case these two parts were indistinguishable, as revealed by immunostaining for α-SMA (Fig. 7D). The endometrium of the *Wnt4^mCh^*^/*mCh*^ uterus did not have the well-developed vasculature system as seen in the wild-type mice (Fig. 7D compare with C, arrowheads). The pan-cytokeratin antibody depicted the luminal epithelium and endometrial glands in the wild-type mice (Fig. 7E), whereas these were missing in the severely malformed uterus of the *Wnt4^mCh^*^/*mCh*^ females (Fig. 7F).

When we analysed the expression pattern of Wnt genes in the 5-month-old mouse uterus by *in situ* hybridization *Wnt4* expression was depicted in the luminal epithelium, endometrial glands and stromal cells of the wild-type uterus (Fig. 7G), but none was present in the *Wnt4^mCh^*^/*mCh*^ mice, since development of the luminal epithelium and endometrial glands had been impaired (Fig. 7H). *Wnt7a* expression is normally restricted to the uterine luminal epithelium (Fig. 7I), but this expression was lost in the *Wnt4^mCh/mCh^* mice (Fig. 7J). Taken together, these observations suggest that ablated *Wnt4* signalling causes defects at very early stages in MD development which lead to further flaws in uterus development and endometrial gland formation.

## Discussion

The mechanism of formation of the MD anlage is still being actively discussed. Although one model has suggested that WD-derived cells contribute to MD growth, lineage tracing experiments have shown that this is not the case (2,4). The WD is needed as a physical guide in the elongation of the MD, however, and it also provides important signals, as demonstrated in *Wnt9b* knock-out mice. These mice have a WD that is the source of *Wnt9b* in the wild-type, but MD elongation is perturbed once *Wnt9*b signalling has been ablated (5). The model that is currently favoured suggests that the MD originates from the CoE, which extends caudally (1,3,4,6,27).

Our time-lapse movies provided evidence that the cell population of the MD primordium indeed originates from the coelomic epithelial cells by migration rather than invagination, in line with earlier studies (3). Our findings indicate that *Wnt4* expression is needed for differentiation of the tip cells and initiation of migration in the MD precursor cells. These cells are guided not only by the WD, but also by signals coming from the CoE. In line with this, we observed that an intact CoE is vital for MD growth, as damage to it prevented MD elongation even when the WD remained intact. Interestingly, the cells of the CoE initiated *Wnt4* expression concurrently with MD elongation, which suggests that certain signals such as *Wnt4* are expressed in a specific spatiotemporal manner and serve to guide MD growth. We also found that the CoE of the *Wnt4^mCh/mCh^* embryos which failed to form any MD had shifted in position and altered in its cell morphology. These observations suggest that the CoE may have an important, but as yet uncharacterized role in controlling MD growth.

Cells of multiple lineages may be needed to construct the MD. Our *Wnt4+* cell-lineage tracing experiments with a dual reporter mouse strain (*mT/mG*) showed that the MD had not only *Wnt4^EGFPCre^*-activated *GFP*+ cells, but also *GFP*− cells, i.e. that multiple cell populations could be involved in forming the MD. The possibility also remains, however, that some cells (in the *Wnt4^EGFPCre^*;*mT/mG* mice) might have been *Wnt4^EGFPCre^ GFP*-negative due to epigenetic silencing of the modified *Wnt4* locus.

The *Wnt4*-positive cells at the tip had cytoplasmic extensions and represented the cells that were leading the migration involved in MD development, while those that migrated behind the leading edge consisted of both Wnt4+ and Wnt4− lineage cells and served to form the duct-like structure. The movies indicated that cells did indeed continue to migrate caudally after the MD primordium had reached the urogenital sinus (1).

The cells that assembled the duct demonstrated epithelial characteristics in that they were located close to each other, they had a limited extracellular matrix, their apical surface was exposed towards the lumen and they had deposited a basement membrane. Nevertheless, despite these numerous morphological epithelial cell features, the embryonic MD at E13.5 in the urogenital ridge did not express typical epithelial cell polarity markers such as cytokeratin-8 or E-cadherin, and it should therefore be referred to as a mesoepithelial duct (2). The tip cells that were derived from the *Wnt4*-positive founder cells became integrated as part of the MD primordium and underwent a mesenchymal-epithelial transition, thereby confirming previous observations that MD tip cells have mesenchymal cell characteristics (4). Our current results demonstrate that the MD tip cells were derived from the *Wnt4*+ founder cell population.

We were also interested in studying whether *Wnt4* is functional after the initiation of the migration of MD-forming cells. An antibody-blocking assay, a useful tool for studying protein functions (28), showed that anti-Wnt4 antibodies did indeed inhibit elongation of the MD. This suggests that the *Wnt4* signal is required not only for the initiation of MD growth, but also for subsequent elongation of the duct. Further evidence of the role of *Wnt4* as a promoter of cell migration was obtained in our *in vitro* scratch-wound-healing assays with NIH3T3 cells that stably expressed *Wnt4* or *Wnt4mCh*. The results indicated that cells expressing *Wnt4* closed the scratch-wound faster than did the controls. The *Wnt4mCh* cells, which represent a hypomorphic *Wnt4* allele, migrated with an intermediate speed relative to *NIH3T3Wnt4* and the controls. Thus, the data support the conclusion that *Wnt4* regulates cell migration and in that way controls MD assembly. Similarly *Wnt4* is also involved in controlling the migration of endothelial and steroidogenic cells (29,30). Moreover, the anti-androgen flutamide that inhibits androgen action did not rescue formation of the MD in *Wnt4*−/− embryos and adult mice (29), a circumstance that is in line with the role of *Wnt4* in controlling MD growth.

*Wnt4* transduces its signalling via both canonical and non-canonical pathways. We used the tankyrase inhibitor XAV939, a molecule that has been successfully used before in an organ culture set-up (31), to inhibit the β-catenin signalling pathway in urogenital ridge explant cultures (26), and found it to have a negative effect on MD formation *in vitro*, causing defects resembling the ones seen in mice with ablated β-catenin signalling (27,32,33). The compound did not inhibit MD growth to any great extent, however. It was also shown that lymphoid enhancer factor 1 (the downstream target of β-catenin signalling) and *Wnt4* signals overlap only in the mesenchyme cells that surround the MD, although *Wnt4* expression appears to be broader, to include the CoE as well (27). All told, this suggests that *Wnt4* could signal via alternative pathways during MD development, a matter that warrants further investigation.

An MD formed in ∼45% of the samples from *Wnt4^mCh/mCh^* hypomorphic mice, but the reduction in the *Wnt4* function caused notable defects in differentiation, including alterations in MD cell polarization, MD tube organization and BM deposition. These findings are in line with our earlier observations that *Wnt4* regulates the organization of granulose cells in ovaries and influences BM deposition in growing ovarian follicles (21).

Our analysis of the uterus in the *Wnt4^mCh/mCh^* females at P21 revealed that the uterine glands had failed to form the myometrium and that the uterus was smaller and dilated due to the formation of a hydro-uterus. Moreover, our previous work had shown a prolonged oestrous cycle in the *Wnt4^flox/flox^; Amhr2^Cre^* conditional knock-out mouse model (21). Fewer uterine glands and a defective decidualization process have been reported in *Wnt4^flox/flox^; PR^Cre^* conditional knock-out mice (34). Our current findings indicate that *Wnt4* is required for the epithelization process both during embryogenesis, when the MD forms, and after birth, when the endometrial glands develop. Similarly, *Wnt4* controls conversion of the mesenchymal pre-tubular aggregates to epithelial vesicles during kidney development (35). The actual detailed molecular mechanism of the mesenchymal-to-epithelial transition in the MD nevertheless remains to be elucidated.

**Figure 8.**
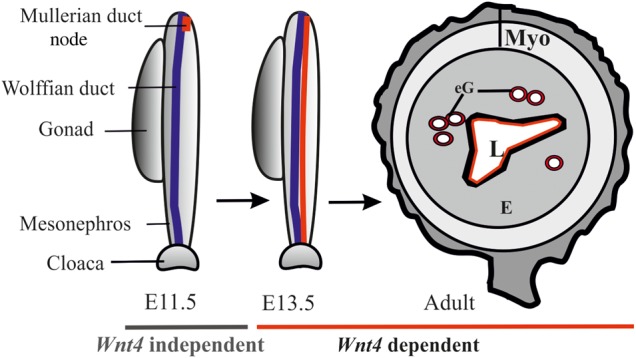
Schematic illustration of the processes regulated by *Wnt4* during female reproductive tract development. *Wnt4* is required during prenatal and postnatal development of female reproductive tract. The initial MD primordium occurs independently of *Wnt4* function (E11.5, the MD primordium in red). After initiation of the process, differentiation of the MD tip cells, prenatal elongation of the MD and postnatal formation of the endometrial gland (eG) all depend on *Wnt4* signalling. The daughter cells that are initially Wnt4+ contribute to MD and eG formation. Myo, myometrium; E, endometrium; eG, endometrial glands; L, lumen.

In summary, *Wnt4* is a crucial factor for the initiation and maintenance of the cell migration that leads to MD formation during female reproductive tract development. In the adult mouse, it is needed for proper myometrium layering and for luminal and endometrial gland formation (Fig. 8). We show that balanced *Wnt4* signalling is constantly needed during embryogenesis, adolescence and adult life to ensure the proper formation and functioning of the female reproductive tract. A deeper understanding of the function of *Wnt4* in female sexual-duct development would help us to explain the pathophysiological mechanisms causing MRKHBL and SERKAL syndromes, endometriosis and infertility.

## Materials and Methods

### Maintenance and genotyping of the transgenic mouse lines

The *Wnt4* knock-out (−/−) (35), *Wnt4^EGFPCre^* (36), *RosaR26R^LacZ^* (Soriano 1999), *RosaR26R^YFP^* (37) and *mT/mG* (38) mice were maintained and genotyped as reported. The generation of the *Wnt4^mCh^*^/+^ knock-in transgenic mice is described below. The transgenic mice were maintained in the *C57Bl/6J OlaHsd* background and littermates were used as controls. All the studies were conducted in accordance with the Finnish national legislation, the European Convention (ETS 123) and EU Directive 86/609/EEC.

### Generation of *Wnt4mCh* knock-in transgenic mice

The *Wnt4mCh* cDNA was produced by polymerase chain reaction (PCR) and targeted to the first exon of the *Wnt4* locus (Supplementary Material, Fig. S4A). Southern blot analysis was used to screen the targeting event and a HindIII-KpnI fragment HK1.3 that corresponds to the nucleotides 2054 and 21773. The 5′ of the homologous arms of the targeting construct was used as a probe for the DNA screening of embryonic stem cells (Supplementary Material, Fig. S4B).

The activity of the fusion protein was analysed in the NIH3T3 cells, and *mCherry*-derived florescence served as an indication of expression (Supplementary Material, Fig. S4C). The *Wnt4mCh* mice were generated in the transgenic core facility at the Biocenter Oulu and genotyped by PCR analysis using DNA isolated from ear clip DNA. The wild-type *Wnt4* allele was identified with the primers 5′-ACTCCTCGTCTTCGCCGTGT and 5′-CAGACGCACTGCCAGCCC, while the *mCherry* allele was depicted with the primers 5′-CGGGAGGCGGCCTTTGTAT and 5′-GGCTTTAGATGTCTTGTTGC. The PCR programme was 94°C for 10 min, 34 cycles at 94°C for 30 s and 58°C for 30 s, followed by an extension at 72°C for 1 min and a final extension step at 72°C for 7 min. Five-percent DMSO was added to the wild-type PCR. The size of the amplicon of the wild-type PCR is 257 bp, while that of the targeted PCR is 593 bp (Supplementary Material, Fig. S4D).

### Histology, immunohistochemistry and β-galactosidase assay

The tissues were dissected in Dulbecco’s phosphate buffered saline (PBS), fixed overnight at +4°C in 4% paraformaldehyde (PFA), washed briefly, dehydrated and embedded in paraffin and sectioned (6 µm). The tissue sections were stained with haematoxylin and eosin or used for immunohistochemistry. Antigen retrieval was enhanced by boiling the slides in 0.02 m citric acid buffer, pH 6, for 20 min or by incubating them in 0.04% pepsin, pH 2, for 30 min. followed by blocking with the appropriate serum for 1 h.

The primary antibodies used were Pax2 (Covance, PRB-276P) (1:100), α-SMA (Abcam ab5694) (1:100) and pan-cytokeratin (1:100) (Santa Cruz, sc-15367). These were incubated overnight at +4°C, washed with PBS, followed by 1 h of staining with the respective Alexa Fluor 488/546 or HRP-conjugated secondary antibody at a dilution of 1:1000. Hoechst 33258 (Polyscience, Inc.) was used to stain the nucleus. The specimens were mounted with Immu-Mount (Fisher Scientific), inspected using a confocal microscope and photographed (Olympus Fluoview FV10-ASW).

The activity of the floxed *RosaR26R*-derived β-galactosidase was identified as published (36), counterstained with eosin for 2 min and mounted with Immu-Mount (Fisher Scientific).

### *In situ* hybridization

The non-radioactive *in situ* hybridization with digoxigenin-labelled RNA probes was performed with the aid of the Intavis AG Bio analytical instrument for the whole mounts, and the BioLane HTI for the slides, as described in (29). The *Wnt4*, *Wnt5a* and *Wnt7a* plasmids required for generating the RNA probes were gifts from A. P. McMahon (Eli & Edythe Broad Center for Regenerative Medicine and Stem Cell Research at USC, USA).

### Organ culture and time-lapse video microscope analysis of the urogenital ridges

The urogenital ridges were micro-dissected from the *Wnt4^EGFPCre^; R26R^YFPflox/+^* and *Wnt4^EGFPCre^; mT/mG* embryos at E11.5 in ice-cold Dulbecco's PBS and placed on Transwell plates (Costar 3450, Corning, Inc.). The tissues were cultured in Dulbecco's modified Eagle's medium (DMEM) 21063 (Gibco) for the time-lapse, whereas DMEM, GlutaMAX (GibcoBRL, Gaithersburg) medium supplemented with 10% foetal bovine serum (FBS), 0.5 mm 4-(2-Hydroxyethyl)piperazine-1-ethanesulfonic acid, N-(2-Hydroxyethyl)piperazine-N′-(2-ethanesulfonic acid) (Sigma, H0887), and 1% penicillin/streptomycin was employed in the antibody-blocking assay. The temperature was maintained at 37°C by means of the TControl Basic 2.3. Program (OkoLab) and a carbon dioxide level of 5% was maintained via the microscope stage incubator (OkoLab). Time-lapse images of the *Wnt4^EGFPCre^; R26R^YFPflox/+^* explants were taken at 20 min intervals with an Olympus XM10 digital camera using the Olympus Cell^P program.

The time-lapse images of the urogenital ridges prepared from the *Wnt4^EGFPCre^; mT/mG* embryos were taken at 5 min intervals with a Zeiss LSM 780 confocal microscope. The novel organ culture set-up used will be described in detail elsewhere. Briefly, the samples were placed on glass cover slips glued to the bottom of a six-well plate and gently compressed by Transwell inserts. In this setting, the images were taken directly through the glass cover slips to obtain the best optical resolution.

We examined time-lapse movies of eight *Wnt4^EGFPCre^*; *mT/mG*, two *mTmG* (control), five *Wnt4^EGFPCre^; R26R^YFPflox/+^* and two *R26R^YFPflox/+^* (control) urogenital ridges.

The antibody-blocking assay was performed with polyclonal anti-Wnt4 goat IgG (R&D Systems) in a concentration of 20 ng/µl, the control specimens being supplemented with 20 ng/µl of goat anti-human IgG antibodies (*Jackson Immuno Research*, Inc.). The tankyrase inhibitor XAV939 (Sigma-Aldrich) was diluted to 10 µm in DMSO (Sigma-Aldrich), and the controls were supplemented with DMSO alone. Urogenital ridges dissected from E11.5 embryos were placed on Transwell inserts in a medium with the antibodies or XAV939 and cultured for 48 h, washed briefly in PBS, fixed in 4% PFA and processed for whole mount *in situ* hybridization.

### Optical projection tomography

Whole mount urogenital ridges from E12.5 embryos were fixed in 4% PFA and stained with Pax2 (Covance, PRB-276P) primary antibody (1:100) followed by washes and staining with the Alexa Fluor 546 secondary antibody (1:800). The specimens were embedded in low-melting point agar, cleared in a mixture of benzyl benzoate and benzyl alcohol (2:1) for 24 h and imaged with an OPT Scanner 3001 m (Bioptonics Microscopy, UK). The OPT data were analysed with the Imaris software (Bitplane, Zurich Switzerland).

### Transmission electron microscopy

The toluidine blue staining and processing of the samples for transmission electron microscopy (TEM) was carried out using the Tecnai GS Spirit Bio Twin microscope (FEI Europe, Edinhoven, Netherlands) as described (39). The images were acquired with a Quemesa CCD camera controlled by the iTEM software (Olympus Soft Imaging Solutions GmbH, Munster Germany).

### Scratch-wound cell migration assay

Mouse embryonic fibroblast NIH3T3 cells, NIH3T3 Wnt4 and NIH3T3 Wnt4mCh cells were used in the *in vitro* scratch-wound assay. 2 × 10^4^ cells/well were seeded onto 96-well plates (Essence Bioscience Image Lock 4379) and grown to confluence. The DMEM 21063 (Gibko) medium was supplemented with 1% penicillin/streptomycin and 10% FBS. A set of cells was incubated in medium containing 0.5 mg/ml of mitomycin (Sigma) for 3 h prior to making the scratch-wound to inhibit cell proliferation. After 24 h of cell culture one central scratch-wound per well was made using the 96-pin WoundMaker (Essence BioScience). After this the cells were grown for a further 24 h and the recovery of the scratch-wound was analysed by taking images at 1 h intervals with an IncuCyte™ automated microscope (Essence BioScience).

The images were analysed with the IncuCyte HD software (Essence BioScience) and the results presented in the form of relative wound densities and standard deviations for each time point. Relative wound density (%) represents the cell density in the wound area expressed relative to that outside the wound area as a function of time. Experiments were performed a minimum of three times in six replicates per experiment the averaged data of experiments is presented (Fig. 2D–G). The percent changes densities were as follows non-treated cells: control 1876%, Wnt4-3T3 1717% and Wnt4mCh-3T3 2068%; mitomycin-treated cells: control 2133%, Wnt4-3T3 1488% and Wnt4mCh-3T3 1764%.

## Supplementary Material

Supplementary Material is available at *HMG* online.

## Funding

This work was supported by grants from the Academy of Finland (206038 and 121647), the Centre of Excellence Grant 2012 – 2017 of the Academy of Finland (251314), the Sigrid Jusélius Foundation and the European Community's Seventh Framework Programme (FP7/2007-2013) under grant agreement FP7-HEALTH-F5-2012-INNOVATION-1 EURenOmics (305608). Funding to pay the Open Access publication charges for this article was provided by Centre of Excellence Grant 2012 – 2017 of the Academy of Finland (284605).

## Supplementary Material

Supplementary Data
