# Supplementary material for: Wnt4 coordinates directional cell migration and extension of the Müllerian duct essential for ontogenesis of the female reproductive tract
Source: Hum Mol Genet. 2015 Dec 31;25(6):1059–73. doi: 10.1093/hmg/ddv621 (PMC4764189; doi:10.1093/hmg/ddv621)
Supplement: Supplementary Data [file supp_25_6_1059__index.html]

Wnt4 Coordinates Directional Cell Migration and Extension of the Müllerian Duct Essential for Ontogenesis of the Female Reproductive Tract — Wnt4 coordinates directional cell migration and extension of the Müllerian duct essential for ontogenesis of the female reproductive tract — Wnt4 coordinates directional cell migration and extension of the Müllerian duct essential for ontogenesis of the female reproductive tract — Supplementary Data 

# Wnt4 coordinates directional cell migration and extension of the Müllerian duct essential for ontogenesis of the female reproductive tract

## Supplementary Data

Supplementary Data

- Supplementary Data - Docx file
