# Supplementary material for: Wnt4 coordinates directional cell migration and extension of the Müllerian duct essential for ontogenesis of the female reproductive tract
Source: Hum Mol Genet. 2015 Dec 31;25(6):1059–73. doi: 10.1093/hmg/ddv621 (PMC4764189; doi:10.1093/hmg/ddv621)
Supplement: Supplementary Data [file supp_ddv621_ddv621supp.docx]

**Supplementary data**

**
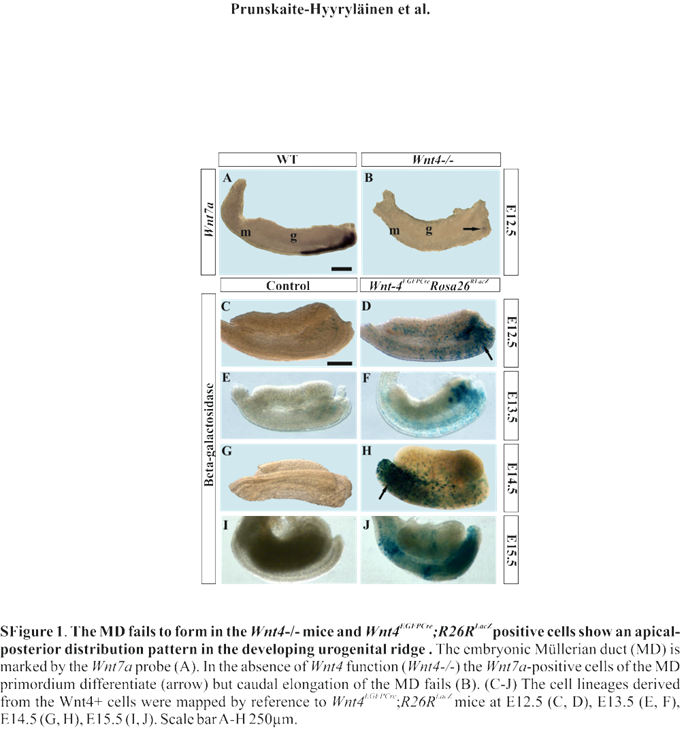
**

**SFigure 1**. **The MD fails to form in the *Wnt4*-/- mice and *Wnt4^EGFPCre^;R26R^LacZ^* positive cells show an apical-posterior distribution pattern in the developing urogenital ridge .** The embryonic Müllerian duct (MD) is marked by the *Wnt7a* probe (A). In the absence of *Wnt4* function (*Wnt4-/-*) the *Wnt7a*-positive cells of the MD primordium differentiate (arrow) but caudal elongation of the MD fails (B). (C-J) The cell lineages derived from the Wnt4+ cells were mapped by reference to *Wnt4^EGFPCre^*;*R26R^LacZ^* mice at E12.5 (C, D), E13.5 (E, F), E14.5 (G, H), E15.5 (I, J). Scale bar A-H 250µm.


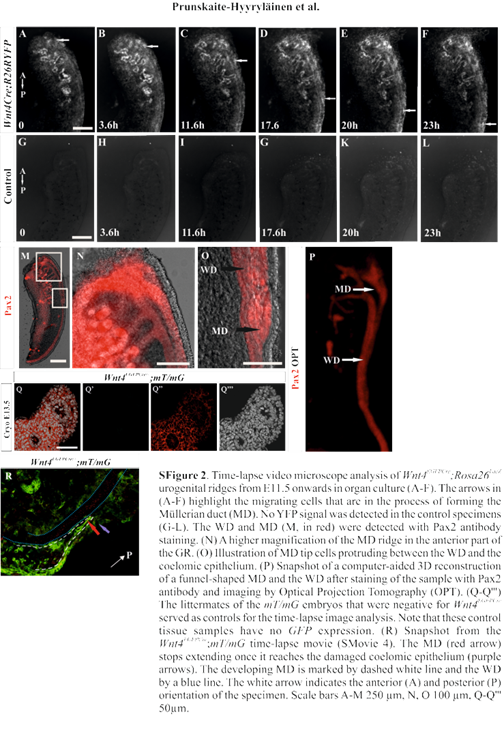


**SFigure 2**. Time-lapse video microscope analysis of *Wnt4^EGFPCre^;Rosa26^LacZ^* urogenital ridges from E11.5 onwards in organ culture (A-F). The arrows in (A-F) highlight the migrating cells that are in the process of forming the Müllerian duct (MD). No YFP signal was detected in the control specimens (G-L). The WD and MD (M, in red) were detected with Pax2 antibody staining. (N) A higher magnification of the MD ridge in the anterior part of the GR. (O) Illustration of MD tip cells protruding between the WD and the coelomic epithelium. (P) Snapshot of a computer-aided 3D reconstruction of a funnel-shaped MD and the WD after staining of the sample with Pax2 antibody and imaging by Optical Projection Tomography (OPT). (Q-Q’’’) The littermates of the *mT/mG* embryos that were negative for *Wnt4^EGFPCre^* served as controls for the time-lapse image analysis. Note that these control tissue samples have no *GFP* expression. (R) Snapshot from the *Wnt4^EGFPCre^*;*mT/mG* time-lapse movie (SMovie 4). The MD (red arrow) stops extending once it reaches the damaged coelomic epithelium (purple arrows). The developing MD is marked by dashed white line and the WD by a blue line. The white arrow indicates the anterior (A) and posterior (P) orientation of the specimen. Scale bars A-M 250 µm, N, O 100 µm, Q-Q’’’ 50µm.

**
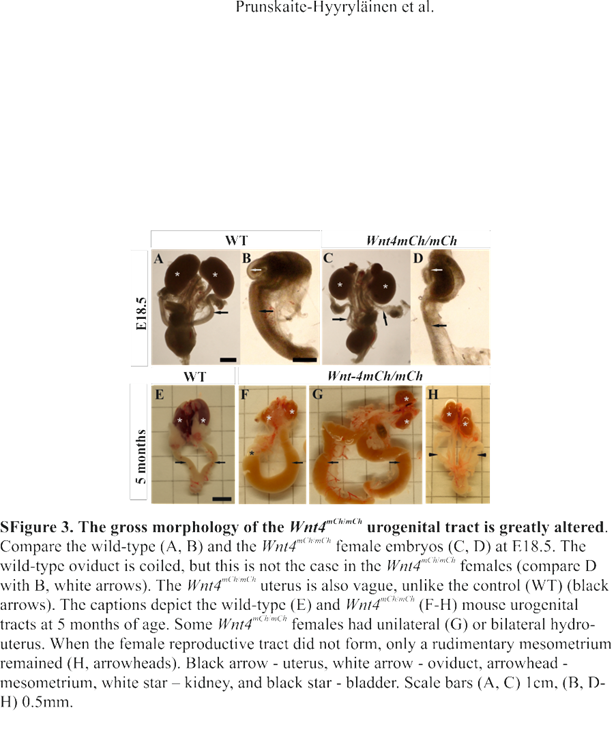
**

**SFigure 3.** **The gross morphology of the *Wnt4^mCh^*^/^*^mCh^* urogenital tract is greatly altered**. Compare the wild-type (A, B) and the *Wnt4^mCh^*^/^*^mCh^* female embryos (C, D) at E18.5. The wild-type oviduct is coiled, but this is not the case in the *Wnt4^mCh^*^/^*^mCh^* females (compare D with B, white arrows). The *Wnt4^mCh^*^/^*^mCh^* uterus is also vague, unlike the control (WT) (black arrows). The captions depict the wild-type (E) and *Wnt4^mCh^*^/^*^mCh^* (F-H) mouse urogenital tracts at 5 months of age. Some *Wnt4^mCh^*^/^*^mCh^* females had unilateral (G) or bilateral hydro-uterus. When the female reproductive tract did not form, only a rudimentary mesometrium remained (H, arrowheads). Black arrow - uterus, white arrow - oviduct, arrowhead - mesometrium, white star – kidney, and black star - bladder. Scale bars (A, C) 1cm, (B, D-H) 0.5mm.

**
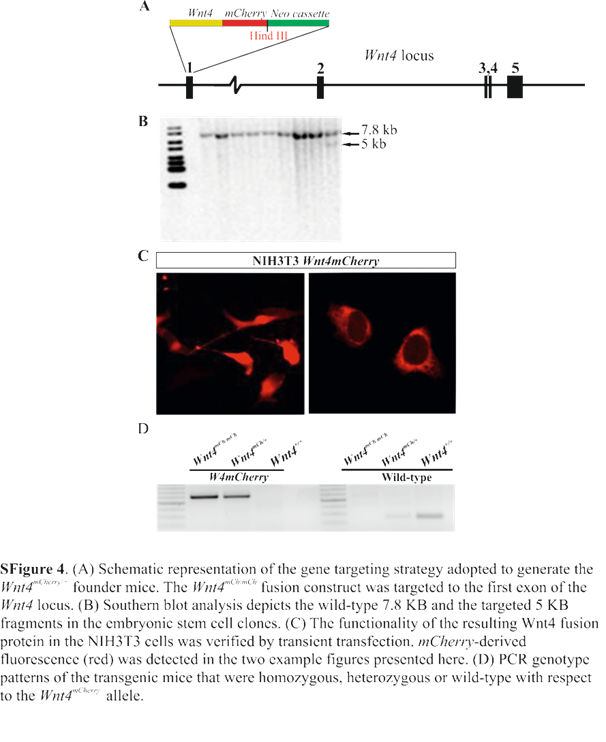
**

**SFigure 4**. (A) Schematic representation of the gene targeting strategy adopted to generate the *Wnt4^mCherry^*^/^*^+^* founder mice. The *Wnt4^mCh^*^/^*^mCh^* fusion construct was targeted to the first exon of the *Wnt4* locus. (B) Southern blot analysis depicts the wild-type 7.8 KB and the targeted 5 KB fragments in the embryonic stem cell clones. (C) The functionality of the resulting Wnt4 fusion protein in the NIH3T3 cells was verified by transient transfection. *mCherry*-derived fluorescence (red) was detected in the two example figures presented here. (D) PCR genotype patterns of the transgenic mice that were homozygous, heterozygous or wild-type with respect to the *Wnt4^mCherry^* allele.

**SMovie 1. The *Wnt4^EGFPCre^R26R^YFP^* cells construct the** **Müllerian duct.** Microscopic time-lapse video of a *Wnt4^EGFPCre^;R26R^YFP^*-positive urogenital ridge that was placed in organ culture at E11.5. Note that the *Wnt4^EGFPCre^*-activated YFP+ cells are located laterally with respect to the Wolffian duct. The cells migrate from the anterior side towards the posterior end and go on to take part in the formation of the Müllerian duct. Selected snapshots from the video are presented in SFigure 2A-F.

**SMovie 2**. **The control *Wnt4^EGFPCre^* species were negative for YFP expression.** The microscopic time-lapse video represents a control wild-type urogenital ridge that was cultured from E11.5 onwards. Note that no YFP-positive cells are seen in the wild-type control. Snapshots from this video are presented in SFigure2 G-L.

**SMovie 3**. **Cells of *Wnt4+* lineage form the MD tip cells and contribute to the differentiation of the Müllerian duct.** The microscopic time-lapse videos of the *Wnt4^EGFPCre^*;*mT/mG* urogenital ridge depict GFP-positive cells activated by *Cre*. GFP is expressed in the coelomic epithelium and in the Müllerian duct primordial cells located on the anterior side of the Wolffian duct (WD) and lateral to it. Note that the GFP-positive tip cells generate protrusions between the WD and the GFP+ coelomic epithelium. The movie represents one ‘out of multiple ‘Z’ planes collected during each time-lapse imaging sequence. Selected snapshots from the video are presented in Figure 1A-D’’’.

**SMovie 4.** **The intact coelomic epithelium is vital for Müllerian duct elongation.** The microscopic time-lapse movie depicts the Müllerian duct as it starts to grow in the *Wnt4^EGFPCre^*;*mT/mG* embryo (red arrow) and then stops. Note that the elongation of the duct is perturbed at the level of the coelomic epithelium, which has been injured mechanically (purple arrow). It is relevant that the Wolffian duct is intact in all the ‘Z’ planes for the entire imaging time and therefore could not be the factor preventing MD elongation. For the markings, see SFigure 2R.
